# Robust Normal Estimation for 3D LiDAR Point Clouds in Urban Environments

**DOI:** 10.3390/s19051248

**Published:** 2019-03-12

**Authors:** Ruibin Zhao, Mingyong Pang, Caixia Liu, Yanling Zhang

**Affiliations:** 1 Institute of EduInfo Science and Engineering, Nanjing Normal Univeristy, Nanjing 210097, China; zhao_rui_bin@163.com (R.Z.); cxsqz@126.com (C.L.); 2 School of Computer Science and Information Engineering, Chuzhou Univeristy, Chuzhou 239000, China; caughy@chzu.edu.cn

**Keywords:** LiDAR point cloud, robust normal estimation, segmentation, urban environments

## Abstract

Normal estimation is a crucial first step for numerous light detection and ranging (LiDAR) data-processing algorithms, from building reconstruction, road extraction, and ground-cover classification to scene rendering. For LiDAR point clouds in urban environments, this paper presents a robust method to estimate normals by constructing an octree-based hierarchical representation for the data and detecting a group of large enough consistent neighborhoods at multiscales. Consistent neighborhoods are mainly determined based on the observation that an urban environment is typically comprised of regular objects, e.g., buildings, roads, and the ground surface, and irregular objects, e.g., trees and shrubs; the surfaces of most regular objects can be approximatively represented by a group of local planes. Even in the frequent presence of heavy noise and anisotropic point samplings in LiDAR data, our method is capable of estimating robust normals for kinds of objects in urban environments, and the estimated normals are beneficial to more accurately segment and identify the objects, as well as preserving their sharp features and complete outlines. The proposed method was experimentally validated both on synthetic and real urban LiDAR datasets, and was compared to state-of-the-art methods.

## 1. Introduction

Light detection and ranging (LiDAR) technology has enabled us to quickly capture three-dimensional (3D) point-cloud data of urban environments, and these data can be used in many applications, such as 3D building reconstruction [1], vegetation-coverage estimation [2], road-marking extraction [3], and digital terrain model (DTM) generation [4]. In these applications, normal estimation plays an important role because many LiDAR data-processing algorithms, including ground filtering [5], object-oriented segmentation [6,7], semantic classification [8,9], and realistic 3D visualization [10], rely on the quality of normal estimation.

### 1.1. Related Work

Normal estimation is a fundamental task in 3D point-cloud processing. In past years, some methods have been proposed for the task, and they can be classified into regression-based methods, Voronoi-based methods, and deep-learning-based methods.

Regression-based estimation was first proposed by Hoppe et al. [11] on the assumption that the surface of an object is smooth everywhere, and the local neighborhood of any point on the surface can thus be approximated well by a plane. For each point in a point cloud, the method first fits a least-squares local plane by performing principal component analysis (PCA) on its neighborhood [12], and then approximately considers the normal of the plane as the normal of the point. Because the method is easy to be implemented and fast to be performed, it is widely used in 3D point-cloud processing, such as point-cloud segmentation [13,14] and denoising [15]. Unfortunately, the method always fails to preserve sharp features of objects because its estimated normals for the points near the sharp features often become overly smooth due to the low-pass property of PCA [16]. In addition, the method is often sensitive to noise and outliers, which are usually contained in 3D point clouds. For this purpose, some techniques were presented to improve the robustness of the method, such as adaptive neighborhood [17,18,19] and weighted distances [20,21]. However, the techniques often require users to manually select parameters in a time-consuming trial-and-error process in order to obtain satisfactory results.

Voronoi-based estimation is based on the construction of a Voronoi diagram [22] for a given 3D point cloud [23]. For each point in the data, Amenta and Bern [24] defined the line through the point and the furthest vertex of the corresponding Voronoi cell as the approximation of the normal at the point. Unfortunately, this method works only for noisefree point clouds [25]. In order to overcome this shortcoming, Dey and Goswami [26] extended the method to deal with noisy point clouds by finding big Delaunay balls, and Alliez et al. [27] proposed a more stable normal-estimation method by combining the advantages of a Voronoi diagram and PCA. Nevertheless, these methods are often incapable of estimating reliable normals for urban LiDAR point clouds because of heavy noise contained in them and their uneven point density.

In the current literature, robust normal estimation has been proven to be effective for accurately processing and analyzing 3D point-cloud data [28,29]. By combining a robust noise-scale estimator and a kernel density estimation, Li et al. [25] proposed a robust normal-estimation method for 3D point clouds of CAD models, and it has the ability to handle noise and sharp features in the models. Boulch et al. [30] proposed a fast and robust method by using a stop criterion to speed the randomized Hough transform and a uniform sampling strategy to overcome the uneven point density of the data. With a low-rank subspace clustering technique, Zhang el al. [31] also presented a robust method based on the observation that accurate normals are easier to obtain for points within smooth regions than points around sharp features. For point clouds of civil infrastructure systems, Khaloo et al. [32] realized a robust normal estimation by discarding outlier points using a high-breakdown multivariate location and scale estimator, and achieved good segmentations of the data with their estimated normals.

Additionally, researchers have begun to estimate normals for unstructured point clouds by using deep learning techniques in recent years. For example, on the basis of the formulation of normal estimation as a discrete classification problem in Hough space, Boulch and Marlet [33] trained a convolutional neural network to predict a normal for each point in an unstructured point cloud according to an image-like accumulator, which is generated from the neighborhood of the point. Moreover, Yizhak et al. [34] also proposed a deep-learning-based method for the normal estimation of point clouds by computing a multiscale point statistics representation for each point in a given point cloud and training a mixture-of-experts network. Similar to the applications of deep learning in other fields, these methods require a large number of training samples with reference normals. Unfortunately, for LiDAR point clouds in urban environments, it is difficult to obtain the reference normals.

### 1.2. Contribution

**Figure 1:**
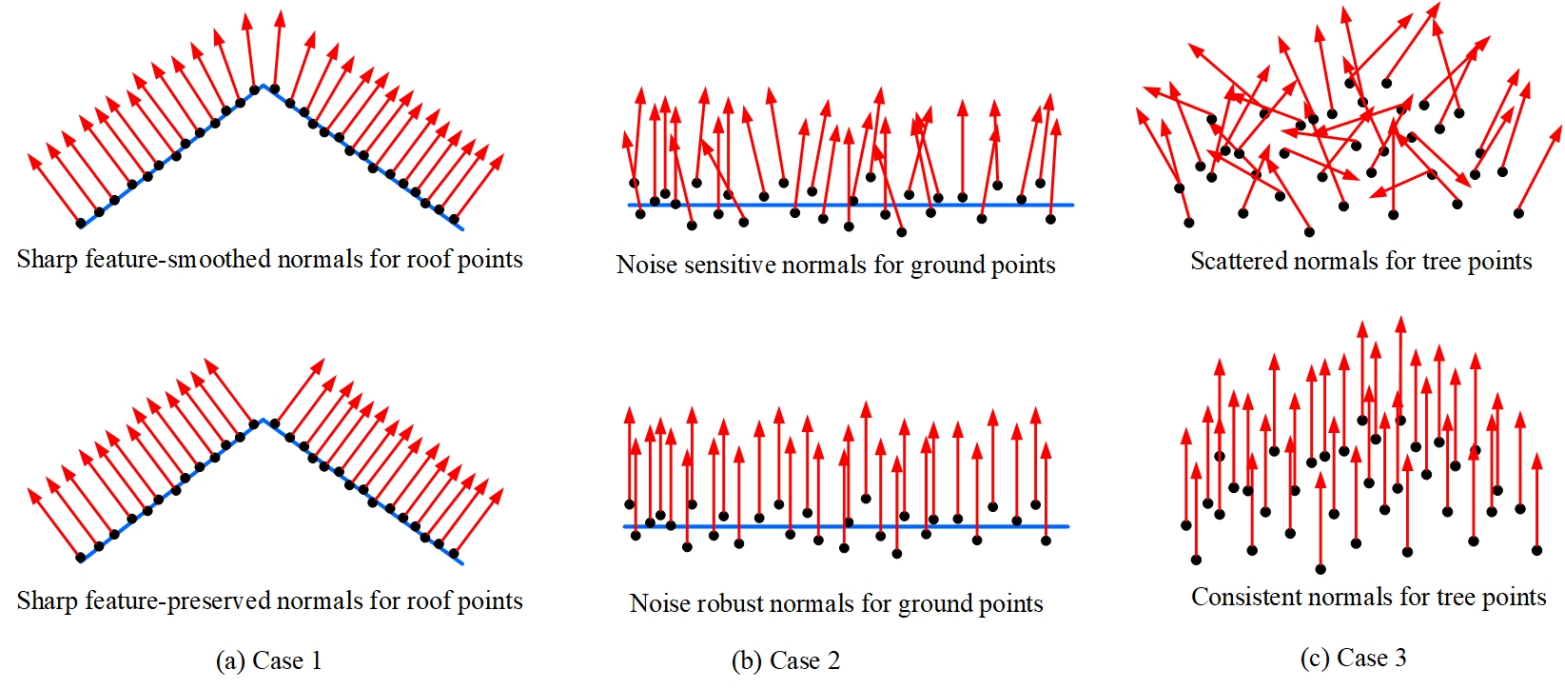
Normal-estimation challenges for light detection and ranging (LiDAR) data of urban environments. Estimated normals for some building points (**left**), ground points (**center**), and tree points (**right**), with the most popular *k*-neighborhood and principal component analysis (KNN–PCA) method (**above**) and the proposed method (**bottom**). Note that the normals of the tree points in Case 3 can be specified on demand by users after identifying them as irregular points in our method.

While there already exist several normal-estimation methods, they were mainly proposed for 3D geometric models (e.g., a mechanical part or an animal character) in the field of computer graphics, and most of the models often have artificial or smooth surfaces as well as low noise. In contrast to a 3D geometric model, a typical urban environment is more complex because it is comprised of numerous different objects, such as buildings, trees, roads, and the ground, and the objects are often varied in their size and surfaces. Furthermore, urban LiDAR data often have a nonuniform density of sampling points and contain a lot of noise. For these reasons, some defects often exist in the normals estimated by using already existing methods. As the three typical cases illustrated in Figure 1, the *k*-neighborhood and PCA-based method (KNN–PCA), which is the most popular normal-estimation method for 3D point-cloud data, always outputs smoothed normals for some edge points of the roofs (Case 1), unreliable normals for noisy ground points (Case 2), and scattered normals for all tree points (Case 3). Clearly, the normals are not conducive to accurately segment the roofs and completely extract the tree and the ground.

From urban environments and their LiDAR point-cloud data, we observed that: (1) objects in environments can be divided into regular and irregular objects, and the surfaces of most regular objects can be approximately represented with a group of local planes; (2) for each point on the object surface with various sizes, selecting a consistent neighborhood to be as large as possible is conducive to reduce noise disturbances and generate a reliable normal; (3) for points on irregular objects such as trees, the corresponding scattered normals estimated conventionally by existing methods would fail to provide meaningful information due to the irregular surfaces of the objects.

Based on the above-mentioned observations, we propose a robust normal-estimation method for urban LiDAR point clouds in this paper. The main contributions of our work are threefold. (1) A group of consistent neighborhoods are generated for points on regular objects in urban environments by robustly detecting plane models at multiscales. Different from *r*-neighborhoods conventionally used in existing methods, consistent neighborhoods allow us to estimate reliable normals with significant noise reduction. (2) The points on irregular objects are robustly identified, which provides better support for users to flexibly specify normals according to their requirements for LiDAR data processing. For example, by specifying a uniform normal to all points on a tree, complete extraction can be obtained for the tree by merging all the points easily using a region-growing algorithm. (3) An accelerating technique and a global optimization technique are presented for fast and accurate normal estimation.

The remainder of this paper is organized as follows. In Section 2, we give a detailed description of the proposed robust normal-estimation method. A group of experiments were performed to evaluate the performance of the method in Section 3, followed by our conclusions in Section 4.

## 2. Methodology

In order to robustly estimate normals for urban LiDAR data, we first determined a group of consistent neighborhoods based on the observation that most of regular objects in urban environments can be described with a group of local planes. Meanwhile, a top–down strategy was employed to make the consistent neighborhoods as large as possible, which is helpful to reduce the impact of noise on the estimated normals. Then, consistent neighborhoods are further refined with a two-step optimization technique, and normals are estimated by performing principal component analysis on the refined consistent neighborhoods.

### 2.1. Consistent Neighbourhood Determination

**Figure 2:**
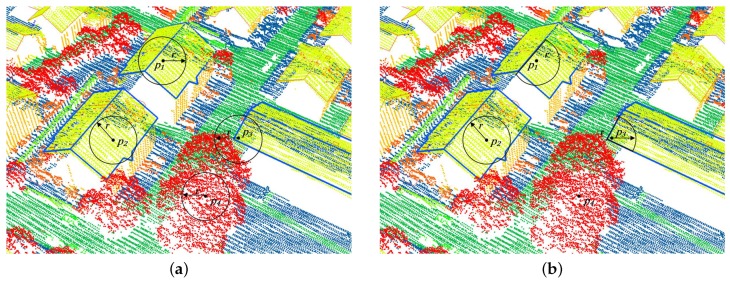
Typical examples of (**a**) *r*-neighborhoods and (**b**) consistent neighborhoods in an urban LiDAR point cloud. Most *r*-neighborhoods in (**a**) are comprised of points derived from different objects, while all consistent neighborhoods in (**b**) are constituted by a group of points sampled from a single object and have good geometric consistency.

For each point $p_{i}$ in a 3D unorganized point cloud, its normal is conventionally estimated from its *r*-neighborhood or *k*-neighborhood. The former contains all points whose distances to $p_{i}$ are equal to or less than *r*, and the latter contains the *k* nearest points to $p_{i}$. In most existing methods, *r* or *k* is often required to be large enough to reduce the impact of noise on the estimated normal. Unfortunately, for urban LiDAR data, a large *r* or *k* tends to cause more inconsistent neighborhoods that consist of heterologous points sampled from different objects. Taking the *r*-neighborhoods shown in Figure 2a as an example, the neighborhood of $p_{1}$ contains a group of points sampled from two building roofs, and the points in the neighborhood of $p_{3}$ are mainly derived from a building roof and a surrounding tree. Obviously, it is difficult to estimate reliable normals for $p_{1}$ and $p_{3}$ simply from the neighborhoods. Contrarily, if a neighborhood contains a group of points sampled from a single object and has good geometric consistency, such as the neighborhoods shown in Figure 2b, it is conducive to estimate a reliable normal.

**Figure 3:**
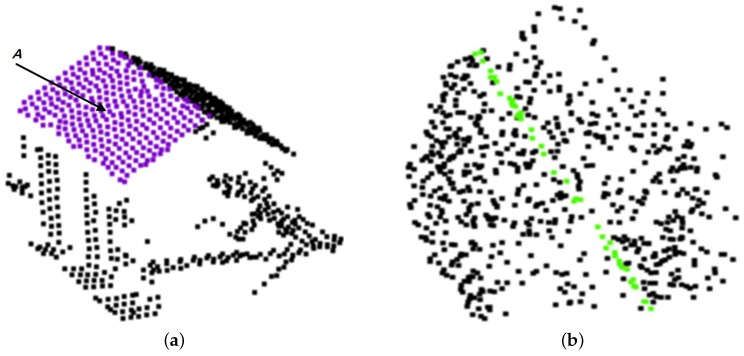
Two plane models originally detected by the RANSAC-based method: (**a**) valid plane for a building roof, and (**b**) spurious plane incorrectly extracted from a group of tree points.

For this purpose, we needed to determine a consistent neighborhood $N_{c,i}$ for each point $p_{i}$ in an urban LiDAR point cloud *P*. Based on the observations of urban environments listed in Section 1, we determined $N_{c,i}$ for $p_{i}$ by detecting a valid plane from the *r*-neighborhood $N_{r,i}$ of $p_{i}$. Here, *r* should be large enough to reduce the impact of noise on the estimated normal. Different from a fixed *r* in existing methods, our method dynamically selects *r* for different points in *P* at multiscales as the method described in Section 2.2. If the points in $N_{r,i}$ are mainly captured from regular objects, one or multiple valid planes may appear in $N_{r,i}$, such as in the neighborhoods of $p_{1}$ and $p_{2}$ in Figure 2a. On the contrary, if most of the points in $N_{r,i}$ are captured from irregular objects, such as the neighborhood of $p_{4}$, no valid plane can usually be found in $N_{r,i}$. In this paper, we employ a random sample consensus (RANSAC) [35] based method to detect the planes in $N_{r,i}$, and determine the consistent neighborhood $N_{c,i}$ for $p_{i}$. The method was proven to be capable of robustly detecting planes from a point cloud that even includes more than 50% outliers. With a predefined distance tolerance threshold δ, the method detects the most probable plane $\hat{M}$ by solving the following optimization problem with an iterative process.

(1) $$\hat{M}=\underset{M}{\arg\;\min}\left\{\sum_{p_{j}\in N_{r,i}}f\left(D\left(p_{j},M\right)\right)\right\}$$

where $D(p_{j},M)$ is the Euclidean distance between $p_{j}$ and *M*, f(·) is an indicator function with $f(D\left(p_{j},M\right)\leq \delta )=1$ and $f(D\left(p_{j},M\right)>\delta )=0$. In each iteration, the method first randomly selects three points from $N_{r,i}$ and determines a plane model *M* from the selected points, and then checks each point in $N_{r,i}$ using the indicator function f(·), and obtains all inner points for *M*. At the end of the iteration, the model that has the most inner points is selected as the most probable plane $\hat{M}$. At the same time, points in $N_{r,i}$ are divided into inner points $N_{r,i}^{\prime }$ and outer points $N_{r,i}^{o}$. The former contains the points whose distances to $\hat{M}$ are equal to or less than δ, and other points in $N_{r,i}$ are contained in the latter. As the most probable plane, $\hat{M}$ has the most inner points among all probable planes. If most of the points in $N_{r,i}$ are mainly sampled from regular objects, $\hat{M}$ is a valid plane, always with the largest area, such as building roof *A* in Figure 3a. On the contrary, $\hat{M}$ is probably a spurious plane if most points in $N_{r,i}$ are derived from irregular objects. Taking the plane with the green inner points in Figure 3b as an example, it is an invalid plane distinctly detected from a group of tree points. Obviously, when a spurious plane is detected, the number of inner points is always smaller than the number of outer points. Therefore, we identify spurious planes with the discriminant rule of $|N_{r,i}^{\prime }\left|>\right|N_{r,i}^{o}|$.

If $\hat{M}$ is a valid plane and $p_{i}$ is contained in $N_{r,i}^{\prime }$, we consider that $N_{r,i}^{\prime }$ is the consistent neighborhood of $p_{i}$, and $p_{i}$ is also considered as a planar point. If $\hat{M}$ is a spurious plane, $\hat{M}$ is ignored and our method tries to detect a valid plane from a small neighborhood of $p_{i}$ with a multiscale strategy given in the next section. The strategy was helpful for us to detect consistent neighborhoods for points on small planar objects or points near the boundaries of planar objects, such as point $p_{3}$ in Figure 2a.

### 2.2. Detect Consistent Neighborhood at Multiscales

An urban LiDAR point cloud often covers a large-scale outdoor scene and contains a great number of objects. The objects typically appear at different spatial scales. Because large neighborhoods are conducive to reduce noise disturbances and generate reliable normals, we used a hierarchical octree to determine a consistent neighborhood as large as possible for each point in the data following a top–down strategy. In addition, an urban LiDAR point cloud often contains a great number of sampling points, and it is time-consuming and redundant to determine a consistent neighborhood for each point in the data. For this reason, only a group of typical points in the data are selected to determine the consistent neighborhoods. The typical points are selected adaptively during the consistent neighborhood detection.

**Figure 4:**
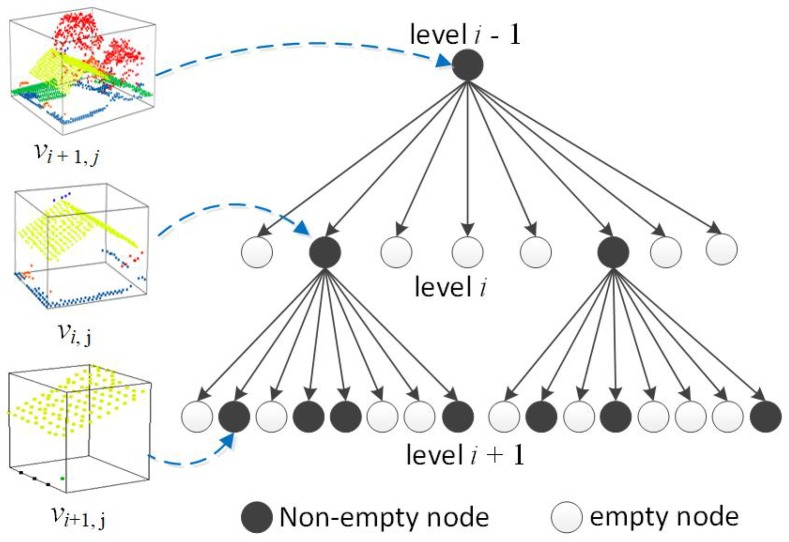
Hierarchical voxelization of the input point cloud with an octree.

At the beginning, input point cloud *P* is voxelized and hierarchically stored into an octree. Taking the whole point cloud as the root node of the octree, voxelization is constructed by recursively subdividing each node into eight child nodes as shown in Figure 4 until the size of the voxels at the lowest level is less than a minimum scale threshold $s_{min}$, which can be specified by referring to the sampling density of *P*. As a result, all points in *P* are divided into a group of voxels which appear at different levels. The higher the levels, the larger sizes the voxels have as well as the more points are likely contained in the voxels. During the hierarchical voxelization, we also obtained some voxels with no or only a few points (such as less than three points), which are regarded as empty voxels in this paper.

On the basis of the generated octree, our method recursively searched each nonempty voxel from the octree, and progressively detected a group of consistent neighborhoods from the input point cloud by using Algorithm 1. With a top–down strategy, the algorithm first detects consistent neighborhoods for large regular objects (e.g., ground surface and large building roofs) at a large scale, and then detects consistent neighborhoods for points on small regular objects at small scales. In Algorithm 1, the current scale is dynamically determined by *r*. For the detected consistent neighbourhood that is capable of containing all points in the current voxel, *r* is defined as $\sqrt{2}\times \left(s_{v}/2\right)$, where $s_{v}$ is the size of the voxel. Additionally, in order to reduce disturbance in the detection process, once a consistent neighborhood is determined, all points in the neighborhood are set to be unavailable for the detection of subsequent neighborhoods.

After performing Algorithm 1 on an input point cloud *P*, a set of consistent neighborhoods $N=\{N_{c,1},N_{c,2},\cdots ,N_{c,k}\}$ are detected for the points in *P*. Each of the neighborhoods in N contains a group of planar points from a single object, the points have good geometric consistency, and the noise in the neighborhoods has been greatly reduced. Obviously, the neighborhoods are helpful for estimating reliable normals for the points in *P* as well as robust to different objects. For large planar objects, such as roads, large building roofs, and the ground surface, consistent neighborhoods are large enough to reduce the impact of noise on the data. For small planar objects, such as small building roofs, consistent neighborhoods can also easily be generated from the voxels at low levels with significantly reduced noise.

**Algorithm 1** Progressively detected consistent neighborhoods at multiscales.
1:**Input**: LiDAR point cloud *P*, octree $O_{t}$, depth of octree $d_{o}$, and distance threshold δ2:**Output**: Consistent neighborhoods $N=\{N_{c,1},N_{c,2},\cdots ,N_{c,k}\}$3:**Initialize**: i←1, N←⌀4:
**while** $i\leq d_{o}$ **do**
5: **for** each voxel $v_{j}$ at the *i*th level of $O_{t}$ **do**6:  Select all available points in $v_{j}$ and calculate their centroid point $p_{c}$7:  Update current radius *r* with size $s_{v}$ of $v_{j}$ as $r=\sqrt{2}\times \left(s_{v}/2\right)$8:  Search *r*-neighborhood $N_{r,c}$ of $p_{c}$ from *P*9:  Detect a plane model $\hat{M}$ from $N_{r,c}$ using Equation (1) with δ10:  Get inner point set $N_{r,c}^{\prime }$ and outer point set $N_{r,i}^{o}$ of $\hat{M}$11:  **if** $N_{r,i}^{\prime }>N_{r,i}^{o}$ **then**12:   Take $N_{r,i}^{\prime }$ as a consistent neighborhood and add it into N13:   Let all points in $N_{r,i}^{\prime }$ be unavailable in the following detection14:  **end if**15: **end for**16: i←i+117:
**end while**



#### Optimization

**Figure 5:**
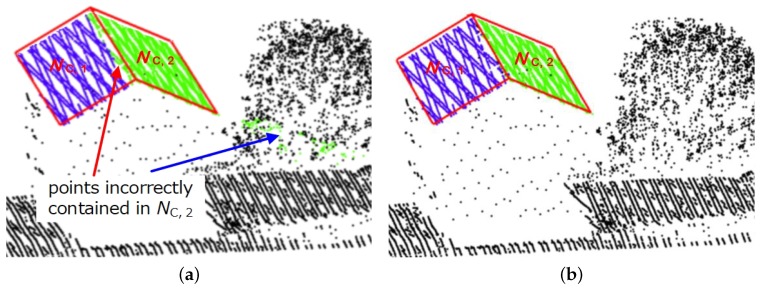
Consistent neighborhoods and their optimization. Consistent neighborhood $N_{c,2}$ in (**a**) contains a few of outer points derived from the neighboring objects, and it is optimized in (**b**) by the proposed technique.

Among all consistent neighborhoods in N, some of them may contain a few outer points, such as nonplanar points from surrounding irregular objects and planar points from other adjacent regular objects. The green consistent neighborhood $N_{c,2}$ shown in Figure 5a is detected intently for the points on the right building roof, whereas it contains a few outer points. Clearly, the points marked by the blue arrow are located on the tree, and the points marked by the red arrow precisely belong to the left building roof, and both of them are mistakenly assigned into $N_{c,2}$ because they are close to $N_{c,2}$ and satisfy plane model $M_{2}$ of $N_{c,2}$ with the predefined distance tolerance threshold δ, i.e., the distances between the points and $M_{2}$ are less than δ.

To address this, our method optimized each consistent neighborhood $N_{c,i}\in N$ with a two-step technique. The first step is used to refine $N_{c,i}$ by filtering out all nonplanar points, such as the green tree points in Figure 5a. In most cases, for a nonplanar point $p_{j}$ that is mistakenly contained in $N_{c,i}$, the majority of its neighboring points are not coincidentally contained into $N_{c,i}$ because of the irregular geometry of the object on which the points are located. On the basis of this fact, we identified $p_{i}$ and removed it from $N_{c,i}$ using the definition: Given $P_{1}=\left\{p_{k}|p_{k}\in N_{r,j}\wedge p_{k}\in N_{c,i}\right\}$ and $P_{2}=\left\{p_{k}|p_{k}\in N_{r,j}\wedge p_{k}\notin N_{c,x}\right\}$, $p_{j}$ is defined as a nonplanar point that is incorrectly contained into $N_{c,i}$ if $|P_{1}\left|<\right|P_{2}|$. Here, $N_{r,j}$ is the *r*-neighborhood of $p_{j}$, $N_{c,x}$ represents any consistent neighborhood in N, and |·| is the cardinalities of $P_{1}$ and $P_{2}$.

The second step is performed to further refine $N_{c,i}$ by reselecting the best-fit consistent neighborhood $\hat{N_{c}}$ for each the planar point $p_{j}$ that is mistakenly contained into $N_{c,i}$, such as the green points marked by the red arrow in Figure 5a. In our method, $\hat{N_{c}}$ is reselected as

(2) $$\hat{N_{c}}=\underset{N_{c}\in N^{\prime }}{\arg\;\min}\left\{\sum_{p_{k}\in N_{r,j}}D\left(p_{k},M\right)\right\}$$

where, $N_{r,j}$ is the *r*-neighborhood of $p_{j}$; $N^{\prime }$ is the set of all candidate consistent neighborhoods, each of which is detected around $p_{j}$ and contains at least one point in $N_{r,j}$; *M* is the plane model of $N_{c}$. If $\hat{N_{c}}\neq N_{c,i}$, we consider that $p_{j}$ should belong to $\hat{N_{c}}$ instead of $N_{c,i}$, and these two neighborhoods are simultaneously optimized by moving $p_{j}$ from $N_{c,i}$ to $\hat{N_{c}}$.

After the two-step optimization technique, all remaining points in each consistent neighborhood $N_{c,i}$ are derived from a single planar object and have a better geometric consistency, such as the points in $N_{c,1}$ or $N_{c,2}$ shown in Figure 5b. In order to maintain high normal-estimation efficiency for massive urban LiDAR data and make all points on a planar object have a group of consistent normals, we consider that all the remaining points in $N_{c,i}$ share a normal, which is estimated by first constructing covariance matrix *C* of the points in $N_{c,i}$ as

(3) $$C=\frac{1}{|N_{c,i}|}\sum_{p_{j}\in N_{c,i}}\left(p_{j}-\bar{p}\right)\times {\left(p_{j}-\bar{p}\right)}^{T}$$

and then selecting the eigenvector associated with the smallest eigenvalue of covariance matrix *C* as the normal for all points in $N_{c,i}$. In the above formula, $\bar{p}$ and |·| are the centroid and cardinality of $N_{c,i}$, respectively.

Besides all the planar points contained into one of the consistent neighborhoods, other points in *P* are always sampled from irregular objects, and all of them are considered as nonplanar points in this paper. On the basis of the identification of nonplanar points, our method supports users to specified normals for the points according to their requirements for LiDAR data processing. Taking tree segmentation as an example, by specifying a uniform normal to all the points, the uniform normal is helpful for meaningfully handling irregular objects, such as extracting a complete tree from *P*. At the same time, the normals have some limitations. For example, they are incapable of distinguishing the points on two nearby trees and rendering them with realistic lighting effects.

## 3. Experiment Results and Performance Evaluation

The performance of our method was evaluated both on synthetic and real datasets, and is compared with two typical existing methods in this section.

### 3.1. Datasets

**Figure 6:**
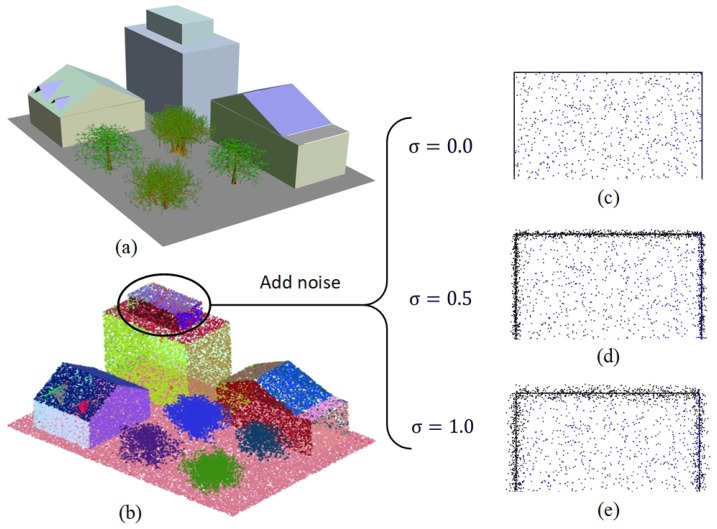
Generation of synthetic LiDAR datasets. (**a**) Simulation scene created within 3D Studio Max software, (**b**) LiDAR dataset synthesized by randomly sampling 100,000 points from the surfaces of the objects in the scene, (**c**,**d**, and **e**) noisy datasets generated by adding different noise scales into the synthesized dataset.

In theory, normal-estimation accuracy can be accurately measured with the angle between the estimated normal and the reference normal of each point in LiDAR data. Unfortunately, reference normals are often unavailable for most real urban LiDAR data. To evaluate the performance of the proposed method, we created a group of synthetic LiDAR datasets as well as their reference normals by modeling a simulation urban scene manually with the 3D Studio Max software. As shown in Figure 6a, the simulation scene contains a group of typical objects in an urban environment, such as buildings, trees, and the ground surface, and the objects varied in size and shape. With the triangular mesh provided by the 3D Studio Max software for the scene, the reference normals can be calculated accurately for each point on the surfaces of the objects. In order to simulate noise in LiDAR data, which are mainly caused by the surface properties (i.e., roughness, reflectivity and small debris) of the real objects in urban environments, and analyze the impacts of the noise on normal estimation, we generated the synthetic datasets with different noise levels by using a two-step process. For each dataset, 100,000 points were first sampled randomly from the surfaces of the objects in the simulation scene, and then a Gaussian noise was added to the points in the direction of their normal as

(4) $$\left(x_{i}^{\prime },y_{i}^{\prime },z_{i}^{\prime }\right)=\left(x_{i},y_{i},z_{i}\right)+n_{i}\times \alpha$$

where $\left(x_{i},y_{i},z_{i}\right)$ is the initial position of point $p_{i}$ in the dataset, and the position is randomly selected on the surfaces of the objects; $n_{i}$ is the reference normal of $p_{i}$ as well as a three-dimensional vector; α is a random number generated by a Gaussian noise generator with mean zero and variance $\sigma^{2}$; and $\left(x_{i}^{\prime },y_{i}^{\prime },z_{i}^{\prime }\right)$ is the noisy position of $p_{i}$ after adding Gaussian random noise to the initial position of the point. The selection of σ determines the noise scale that is added into the dataset. In our experiment, by specifying σ as 0.0, 0.25, 0.5, 0.75, and 1.0, we generated five noisy synthetic datasets that had different noise levels. From the examples shown in Figure 6c–e, we could clearly find that the noise scale becomes increasingly larger with the increase of σ.

**Figure 7:**
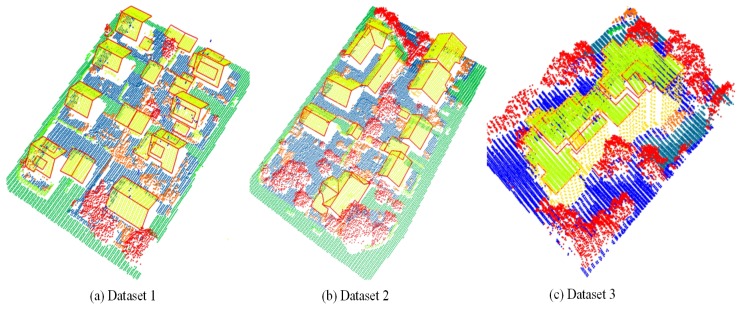
Three real LiDAR datasets for evaluating the performance of our method. The points are rendered according to their manual labels, and the red lines are the reference boundaries of the roofs.

Besides the synthetic datasets, three real LiDAR datasets were also involved in our experiments. As shown in Figure 7, the objects, especially buildings and trees, in the three datasets are different. For example, some buildings in dataset 1 have a group of inconspicuous roofs characterized by small size or fuzzy boundaries, and the building in dataset 3 is a large and complex historic building surrounded by trees. Meanwhile, point density was also different among the three datasets. Specifically, both dataset 1 and dataset 2 had a point density of about four points/$m^{2}$, while dataset 3 had a point density of about eight points/$m^{2}$. All three datasets were derived from the airborne laser-scanning data captured over Vaihingen in Germany and used for the urban classification and 3D reconstruction benchmark of the International Society of Photogrammetry and Remote Sensing (ISPRS) [36]. Each point in the datasets was manually labelled by Niemeyer et al. [37] as one of nine categories: powerline, impervious surface, low vegetation, car, fence/hedge, roof, facade, shrub, and tree, which were distinguished with different colors in Figure 7. Among the categories, impervious surface, roof, facade, and low vegetation were considered as regular objects in our method because their surfaces can be described with a group of local planes, and other categories of the objects were considered as irregular objects or small objects. In addition, the reference boundaries of each roof were provided by ISPRS, and they are visualized with red lines in Figure 7. The manual labels of the points and the reference boundaries of the roofs are useful for us to quantitatively evaluate our results.

### 3.2. Competing Methods and Parameter Selection

Two typical normal-estimation methods, KNN–PCA and the randomized Hough transform-based method (RHT), were involved in our comparison. As the most popular method, KNN–PCA is simple and efficient, and has been widely used in 3D point-cloud processing [13,14,15]. The method estimates normals for each point by performing PCA on the *k*-neighborhood of the point. In this method, the selection of *k* has a significant influence on normal estimation. In most cases, a large *k* is helpful in reducing disturbance from noise in the data, but it also causes some sharp features of objects in the data to be smoothed out. In our experiment, we used the implementation of the method from the point cloud library [38].

The RHT-based method was proposed as a fast and robust normal-estimation method for 3D point clouds [30]. In the method, the filled Hough transform accumulator is considered as an image of the discrete probability distribution of possible normals, and it estimates the normals corresponding to the maximum of this distribution. The method is robust to noise in point-cloud data, and has the ability to preserve the sharp features of objects in the data. In the method, four main parameters, number of primitives $T_{R}$, number of neighbors *k*, number of bins $n_{\phi }$, and number of accumulator rotations $n_{rot}$, have a significant impact on the accuracy and efficiency of the method.

In our method, two parameters, δ and $s_{min}$, need to be specified according to the surface roughness and sampling density of the given urban LiDAR dataset. The parameter δ is used to define the distance tolerance threshold for detecting consistent neighborhoods, and it can be specified according to the surface roughness of the regular objects. The rougher the surface of the objects, the larger δ should be. The parameter $s_{min}$ is the minimum scale for detecting consistent neighborhoods, it determines the size of the voxels at the lowest level of the octree.

For the synthetic and real datasets in our experiment, the parameters in the three methods were manually selected for obtaining the best possible result, and we listed the selected values for the parameters in Table 1.

### 3.3. Experiment Results and Discussion

#### 3.3.1. Synthetic Datasets

Based on the reference normals of the synthetic datasets, we evaluated the performance of the three methods by using two measures, root mean square (RMS, as Equation (5)) and root mean square with threshold ($RMS_{\tau }$, as Equation (6)), which were specially designed for evaluating the accuracy of normal estimation [30,31]. The two measures were defined as:

(5) $$RMS=\sqrt{\frac{1}{\left|p\right|}\sum_{p_{i}\in P}{\hat{n_{p_{i},ref},n_{p_{i},est}}}^{2}}$$

(6) $$RMS_{\tau }=\sqrt{\frac{1}{\left|p\right|}\sum_{p_{i}\in P}{v_{p}}^{2}}$$

where

(7) $$v_{p}=\left\{\begin{array}{cc} \hat{n_{p_{i},ref},n_{p_{i},est}} & \mathrm{if}\hat{n_{p_{i},ref},n_{p_{i},est}}<\tau \\ \pi /2 & \mathrm{otherwise} \end{array}\right.,$$

where $n_{p_{i},ref}$ and $n_{p_{i},est}$ are the reference normal and estimated normal of point $p_{i}$ in given point cloud *P*, $\hat{n_{p_{i},ref},n_{p_{i},est}}$ is the angle between $n_{p_{i},ref}$ and $n_{p_{i},est}$, and τ is an angle error threshold. RMS is often used to measure the average error of the estimated normals for all points in *P*. However, it always hides the large errors of the normals that are frequently estimated for edge points. Although these points are a minority in urban LiDAR data, they often play an important role in accurate data segmentation or classification. For the purpose, we also used $RMS_{\tau }$ in our evaluation. In contrast with RMS, $RMS_{\tau }$ is sensitive to bad normals that have an error above τ by specifying a very bad error of π/2 to each of them.

With the parameters listed in Table 1, we estimated normals for the five synthetic datasets by separately using the three methods, and listed the results in terms of RMS, $RMS_{\tau }$, and the percentage of the bad normals (β) in Table 2. The results show that the KNN–PCA-based method obtained a group of small RMS for the five datasets, which indicate that the method is capable of estimating accurate normals for most points in the datasets. However, the largest $RMS_{\tau }$ and β were also obtained by the method for all the datasets with various noise levels, which means that the results of the method include most bad normals as defined in Equation (6). In addition, both $RMS_{\tau }$ and β of the method rose significantly with the increase of noise levels, which indicates that the results of the method were sensitive to noise in the datasets. Between the three methods, our method obtained the smallest RMS as well as the smallest $RMS_{\tau }$ and β for almost all of the datasets, and all measures had a very slow growth rate with the increase of noise levels. This means that our method not only estimated the most accurate normals for almost all points in the datasets, but also had better robustness against noise than the two other methods.

**Figure 8:**
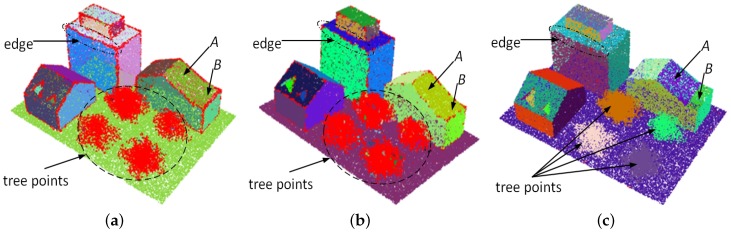
Segmentation results of one of synthetic datasets according to the normals estimated by (**a**) KNN–PCA, (**b**) randomized Hough transform RHT method, and (**c**) the proposed method, respectively.

A reliable estimation of normals is always conducive to accurately segment urban LiDAR data. For this reason, we further evaluated the performance of the methods by segmenting the synthetic dataset according to the normals estimated for it by using the methods. In this paper, the region-growing algorithm [39] implemented in the point cloud library [38] was used, which segments the dataset by progressively merging the points with similar normals into a cluster under the constraints of two predefined parameters, normal tolerance threshold $n_{k}$, and the minimum number $n_{p}$ of points for a valid cluster. On the basis of the most accurate normals, i.e., the normals with the smallest $RMS_{\tau }$, estimated by the three methods, we segmented the synthetic dataset separately by using the region growing algorithm with $n_{k}=6^{{}^{\circ}}$ and $n_{p}=15$. The results were illustrated in Figure 8, where each color represents a cluster segmented from the dataset by using the algorithm and all residual points were colored in red. As shown in Figure 8a,b, many edge points were not effectively handled in the results of KNN–PCA and RHT because of the unreliable normals estimated by the two methods. Meanwhile, building roofs *A* and *B* also failed to be distinguished due to the inconspicuous boundary between them. From the result shown in Figure 8c, we could find that almost all points in the dataset were correctly handled, and building roofs *A* and *B* were effectively distinguished by using the normals estimated by our methods. In addition, all tree points in the dataset were meaningfully divided into four blocks by specifying a group of consistent normals for them in our method. However, because both KNN–PCA and RHT estimate a group of scattered normals for points on irregular objects, the tree points were not meaningfully handled in Figure 8a,b.

#### 3.3.2. On Real Datasets

**Figure 9:**
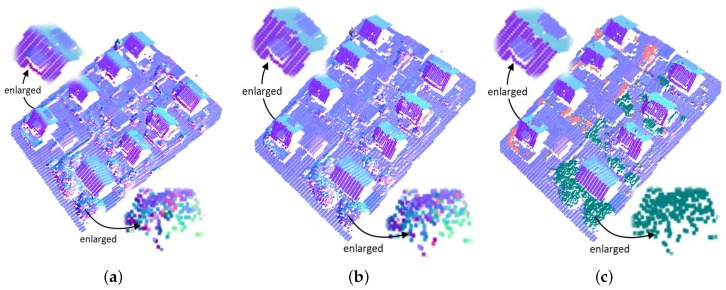
Normals estimated for a real LiDAR dataset by using (**a**) KNN–PCA, (**b**) RHT, and (**c**) our method, respectively.

With the parameters listed in Table 1, we estimated normals for the three real LiDAR datasets by using KNN–PCA, RHT, and our method, respectively. Taking the estimated normals for Dataset 1 as an example, we illustrated the results of the three methods in Figure 9, where the normals were converted into the hue-saturation-value (HSV) color space to clearly display them. As shown in Figure 9a,b, the normals estimated by KNN–PCA and RHT, for the points near the boundaries of the building roofs were undesirably smoothed, which can easily be found from the smooth color transitions along the edge points of the building roofs enlarged in the figure. In addition, both KNN–PCA and RHT, produced a group of scattered normals for the points on irregular objects, such as the points on the tree enlarged in the figure. Obviously, normals are not reliable enough to accurately segment the building roofs and completely extract the tree from the LiDAR data. Conversely, in our result shown in Figure 9c, the boundaries of the building roofs are clearer and more complete, and the estimated normals for the tree points are more consistent.

**Figure 10:**
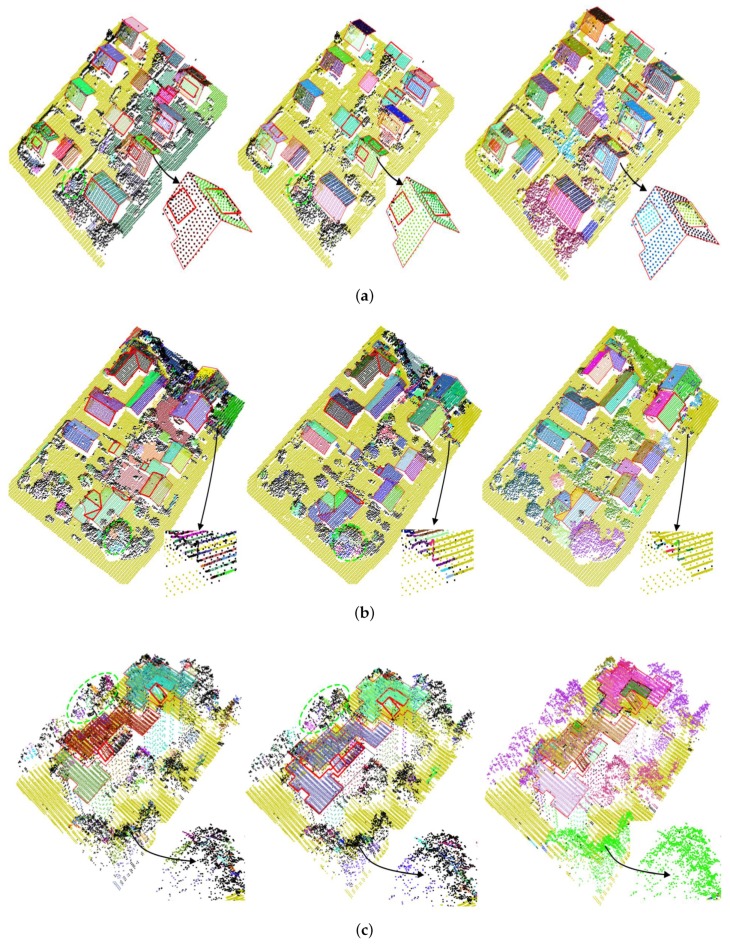
Segmentation results of three real LiDAR datasets in urban environs. (**a**) Dataset 1, (**b**) dataset 2, and (**c**) dataset 3. From left to right, the results of each dataset were generated with the normals estimated by using the KNN–PCA-based method, the RHT-based method, and the proposed method, respectively. In this figure, the undetected building roofs are marked by bolded polygons.

Because the reference normals of the real datasets are unavailable and difficult to obtain, we did not evaluate the accuracies of the estimated normals by using measures RMS and $RMS_{t}$, defined in Section 3.3.1. Instead, we first segmented the three datasets based on the normals estimated by KNN–PCA, RHT, and our method, respectively, and then indirectly evaluated the performance of the three methods by analyzing the impact of the normals on dataset segmentation. Similar to the synthetic dataset used in Section 3.3.1, the three real datasets were also segmented by employing the region-growing algorithm [39] in this experiment, with $n_{k}=6^{{}^{\circ}}$ and $n_{p}=30$. All segmentation results are illustrated in Figure 10, where the resulting clusters were displayed with different colors, all residual points were colored in black, and the reference boundaries of the roofs were visualized with the red polygons.

As shown in Figure 10, the estimated normals by using different methods led to different segmentation results for the datasets. From the first two columns of Figure 10, we could find that the normals estimated by KNN–PCA and RHT, tend to cause the following defects: (1) Some inconspicuous roofs failed to be detected, such as roofs marked by the bolded polygons in Figure 10 and the typical examples of the roofs enlarged in Figure 10a. The main reason for the failures is that the normals estimated by KNN–PCA and RHT, for the points on the roofs are not accurate due to the small sizes or fuzzy boundaries of the roofs. (2) Many meaningless clusters were generated from points on irregular objects, such as the clusters marked by green circles in Figure 10c. In addition, some points on regular objects were also divided into a group of meaningless clusters due to uneven point density or sharp features of the objects, such as the ground points enlarged in Figure 10b. (3) There were many residual points in the segmentation results. All the residual points were displayed with black in Figure 10, and most of them were located on irregular objects, especially trees, or on roof boundaries.

In contrast, the three datasets were more accurately segmented with the normals estimated by our method. As the results show in the last column of Figure 10, almost all of the points, including the points on the trees and on the boundaries of the building roofs, were divided into a group of meaningful clusters. Meanwhile, the number of the undetected roofs have been significantly reduced. In Table 3, we have listed three measures, $n_{r}$, $n_{b}$, and $n_{t}$, of the segmentation results obtained by using the three methods for the datasets. $n_{r}$ is the number of the undetected roofs, $n_{b}$ is the number of residual building points, and $n_{t}$ is the number of residual tree points. All of them are sensitive to the accuracy of the segmentation results. As listed in Table 3, the minimum $n_{r}$, $n_{b}$, and $n_{t}$ were obtained for all the three datasets with the normals estimated by using our method.

## 4. Conclusions

The three-dimensional LiDAR point clouds of urban environments typically contain a great number of complex and various objects, as well as kinds of noise. For this reason, the normals estimated by existing methods are often not reliable enough to accurately process the data. Inspired by the observation that an urban environment comprises regular objects and irregular objects, and the former can often be represented by a group of local planes, in this paper we robustly estimated normals for an urban point cloud by detecting valid planes at a multiscale and determining a group of consistent neighborhoods. The experiments on synthetic and real datasets reveal that the estimated normals are reliable, and also helpful for segmenting urban LiDAR data accurately and meaningfully.

At present, the normals estimated by the proposed method are unoriented, which means that the points on a planar object (e.g., a building roof) may have a group of normals with opposite directions. In our experiments, we used the algorithm implemented in the point cloud library [38] to reorient the normals. By specifying a viewpoint high enough above the ground, the algorithm is capable of producing a group of consistently oriented normals in most cases. However, the algorithm generates some unexpected results while dealing with the point clouds that contain some downward-facing objects. The reason is that the algorithm results are sensitive to the selection of the viewpoint. In future work, we will investigate a robust reorienting method. Furthermore, some urban environments may contain a few regular objects with curved surfaces, so we also intend to extend our method to deal with these objects. 

## Figures and Tables

**Table 1:** Manually selected parameters for three methods to estimate the normals of the synthetic and real datasets. Note: RHT is the randomized Hough transform-based method.

Datasets	KNN–PCA	RHT	Proposed Approach
Synthetic datasets	k = 50	$T_{R}$ = 50, k = 20, $n_{\phi }$ = 5, $n_{rot}$ = 15	δ = 0.15, $s_{min}$ = 4
Real datasets	k = 30	$T_{R}$ = 80, k = 40, $n_{\phi }$ = 5, $n_{rot}$ = 15	δ = 0.10, $s_{min}$ = 3

**Table 2:** Root mean square (RMS), $RMS_{\tau }$, and β of the normals estimated by using the *k*-neighborhood and PCA-based method (KNN–PCA), RHT, and the proposed approach, respectively, for synthetic datasets.

Noise (μ)	KNN–PCA			RHT			Proposed Approach		
	RMS	${\mathrm{RMS}}_{\tau }$	β (%)	RMS	${\mathrm{RMS}}_{\tau }$	β (%)	RMS	${\mathrm{RMS}}_{\tau }$	β (%)
0.00	0.152	0.406	7.98	0.200	0.210	2.14	0.147	0.154	1.16
0.25	0.156	0.414	8.28	0.193	0.234	2.65	0.148	0.157	1.20
0.50	0.171	0.443	9.47	0.192	0.234	3.62	0.154	0.162	1.27
0.75	0.194	0.605	17.52	0.195	0.319	4.87	0.169	0.185	1.40
1.00	0.225	0.785	29.59	0.204	0.360	6.22	0.171	0.171	1.58

**Table 3:** Statistics about the segmentation results of real datasets. $n_{r}$, $n_{b}$, and $n_{t}$ are the number of undetected roofs, the number of residual building points, and the number of residual tree points, respectively.

ID	KNN–PCA			RHT			Proposed Approach		
	$n_{r}$	$n_{b}$	$n_{t}$	$n_{r}$	$n_{b}$	$n_{t}$	$n_{r}$	$n_{b}$	$n_{t}$
Dataset 1	14	770	1054	9	279	1044	2	138	45
Dataset 2	11	3187	5303	4	826	4480	1	313	306
Dataset 3	2	587	6091	5	91	5676	0	22	147
